# 160 Clinical Practices of Clostridioides difficile Testing, Symptom Assessment, and Contact Precautions in a Tertiary-Care Hospital

**DOI:** 10.1017/ash.2026.10561

**Published:** 2026-06-23

**Authors:** Huong Thu Thi Dinh, Cap Nguyen Trung, Hoa Le Nguyen Minh, Trang Thi Nguyen, Tam Thi Pham, Hue Nguyen Thi

**Affiliations:** 1 National Hospital of Tropical Diseases; 2 National Hospital Tropical Diseases, Hanoi, Viet Nam

## Abstract

**Background:** Healthcare-associated bloodstream infections (HABSIs) are major cause of morbidity and mortality in intensive care units (ICUs), particularly in low - and middle-income countries (LMICs). However, ICU-level surveillance data describing the epidemiology and antimicrobial resistance pattern of bloodstream infections in Vietnam remain limited. **Methods:** We conducted a restrospective descriptive study using routine infection prevention and control (IPC) surveillance data from an adult ICU at a tertiary infection diseases hospital in Hanoi, Vietnam. All laboratory-confirmed HABSI episodes recorded between October 2023 and December 2025 were included. Cases were identified using standardized national IPC surveillance definitions aligned with CDC/NHSN principles. Demographic characteristics, exposure to invasive devices, microbiological profiles and antimicrobial resistance patterns were analyzed. **Results:** A total of 134 HABSI episodes were identified. Male patients accounted for 73.9% (99/134) of cases. Most infections occurred among critically ill ICU patients with prolonged ICU stays and exposure to invasive devices. Central venous catheters were present in the majority of HABSI episodes 84.3% (113/134), suggesting a high level of exposure to intravascular devices. Gram-negative organisms predominated (82 episodes) with Acinetobacter baumannii was the most frequently identified pathogen (20.9%), followed by Klebsiella pneumoniae (15.7%) being the most frequently isolated pathogens. A high proportion of isolates demonstrated multidrug resistance, with 67.1% (55/82) of gram -negative isolates exhibiting carbapenem resistance, predominantly a mong A.baumanii and K.pneumoniae . In addition, fungal bloodstream infections due to Candida species accounted for 30 episodes (22.4%). **Conclusion:** HABSI in this adult ICU were associated with frequent central venous catheter use and a high burden of antimicrobial resistance, particularly carbapenem-resistant gram-negative bacteria dominated by Acinetobacter baumanii. The substantial proportion of candidemia further highlights the complexity of bloodstream infections in critically ill patients. These findings underscore the importance of strengthening facility-based IPC surveillance, optimizing central line care practices, and integrating stewardship interventions in high-risk ICU settings in Vietnam.

